# Molecular Transmission Dynamics of HIV-1 in Migrant Populations: Transmission Clusters and Demographic Diversity in Hangzhou, a Key Migration Hub in Eastern China

**DOI:** 10.3390/v18030365

**Published:** 2026-03-16

**Authors:** Sisheng Wu, Ling Ye, Xingliang Zhang, Min Zhu, Wenjie Luo, Zhou Sun, Junfang Chen, Ke Xu

**Affiliations:** 1 Hangzhou Center for Disease Control and Prevention (Hangzhou Health Supervision Institution), Hangzhou 310021, China; wuss543@foxmail.com (S.W.); lqyeling@126.com (L.Y.);; 2 Zhejiang Key Laboratory of Multi-Omics in Infection and Immunity, Hangzhou 310021, China

**Keywords:** HIV-1, migrant populations, molecular transmission network, MSM population, molecular epidemiology

## Abstract

Objective: Population mobility complicates the prevention and control of HIV. To address these challenges, this study explored the molecular epidemiology of HIV among migrant populations in Hangzhou. Methods: People newly diagnosed with HIV/AIDS from 2020 to 2023 were divided into permanent migrant population (PMP), temporary migrant population (TMP), and non-migrant population (NMP). HIV-1 *pol* gene sequencing was performed to calculate genetic distance. Sample pairs with genetic distances ≤0.005 were used to construct the molecular transmission network. Results: PMP comprised people living with HIV in Hangzhou, characterized by younger age, higher education, and predominantly homosexual transmission. This population forms multiple large molecular clusters together with NMP. TMP accounted for the highest proportion of females and people infected through heterosexual contact, but the education level was the lowest. NMP had the fewest people living with HIV. The main subtypes identified were CRF01_AE, CRF07_BC, CRF08_BC and CRF55_01B. Drug resistance prevalence did not differ significantly among the populations. The molecular transmission network included 833 cases forming 275 clusters, with an overall sample inclusion rate of 23.04%. PMP, TMP and NMP inclusion rates were 27.10%, 19.03% and 21.4%, respectively. All molecular clusters involved migrant populations. Factors associated with inclusion in the network for migrants included current residence, household registration, STD history, sample source, and stage at diagnosis. Conclusions: Migrant populations play a major role in ongoing HIV transmission. Prevention and control measures should be strengthened according to population-specific characteristics. Molecular transmission networks are useful tools for assisting precise control.

## 1. Introduction

Hangzhou, the capital of Zhejiang Province and a central city in the Yangtze River Delta region, is the primary destination of migrant populations in Zhejiang Province. Data from the seventh national census indicate that Hangzhou attracts 18% of inter-provincial migrants and 75% of intra-provincial migrants to Zhejiang [1]. While migrant populations contribute to urban development and construction, they also present challenges to HIV/AIDS prevention and control. Sexual transmission had been the main channel of HIV transmission in Hangzhou [2]. Migrants engage in a considerable level of unprotected sexual behavior, presenting a significant HIV risk [3,4]. Their knowledge of infectious disease prevention is often insufficient, and they are prone to being infected with diseases [5]. If infected with HIV, their high mobility and broad activity range can expand transmission scope.

The HIV genome is highly variable, and its genetic sequence undergoes constant change within the host due to selection pressure [6,7]. Genetic distances derived from the gene sequence reflect sequence similarity and viral relatedness [8]. Through the topological mapping of these distances, cases with similar sequences cluster together, and all clusters form the molecular transmission networks [9,10]. The molecular transmission networks provide a visual representation of HIV molecular epidemiological patterns, help warn outbreaks and identify HIV transmission chains. They allow public health workers to target limited prevention resources more effectively. The large volume of sequences generated from HIV drug resistance testing has enabled countries, including China, the United States, and Bulgaria, to use these data for constructing molecular transmission networks and monitoring transmission dynamics [11,12,13,14,15].

The mobility of migrant populations who temporarily live in Hangzhou was stronger than those who permanently live in Hangzhou. The follow-up methods of the two groups are different: temporary migrant population receives follow-up at their household registration, while permanent migrant population receives follow-up therapy in Hangzhou. Therefore, in this study, based on the degree of population mobility, the people newly diagnosed with HIV were classified into permanent migrant population (PMP), temporary migrant population (TMP), and non-migrant population (NMP). We built the molecular transmission network in Hangzhou to analyze and compare the molecular epidemiological characteristics among the three groups, aiming to facilitate the precise prevention and control of HIV.

## 2. Materials and Methods

### 2.1. Study Population and Sample Collection

Plasma samples were collected from people newly diagnosed with HIV before receiving antiviral therapy in Hangzhou from 2020 to 2023. The inclusion criteria for the samples were as follows: (1) cases reported in Hangzhou; (2) the successful acquisition of HIV sequence; and (3) informed consent of the patient. Demographic and epidemiological information (e.g., age, gender, ethnicity, education, current residence, household registration, infectious route, and other HIV infection-related information) was obtained through face-to-face surveys.

In this study, people living with HIV were classified into permanent migrant population (PMP), temporary migrant population (TMP) and non-migrant population (NMP). PMP was the population whose current residence was Hangzhou but whose household registration was not. TMP was the population whose current residence and household registration were both not Hangzhou. NMP was the population whose current residence and household registration were both Hangzhou. The determination of the infectious stage at the time of diagnosis in this study was performed as follows: Early stage: People at this stage were infected with HIV within\6 months, and people had a high viral load and incomplete antigen–antibody reaction. The early stage should meet one of the following conditions: (1) the baseline viral load is greater than 10^6^ CPs/mL with positive serologic testing results and (2) the positive nucleic acid testing plus negative or indeterminate serologic testing results, and the patient is not in the late stage [16,17]. Samples in the early stage were not verified by Lag-Avidity, i.e., determination may be erroneous [18]. The late stage, also known as the AIDS stage, is determined according to the diagnosis and treatment guidelines [19]. Intermediate stage involves a person living with HIV who is neither in the early stage nor the late stage.

### 2.2. Nucleic Acid Extraction and Sequence

HIV-1 RNA was extracted from plasma samples using QIAamp Viral RNA Mini Kit (QIAamp, Hilden, Germany). The viral *pol* gene (including the full length of protease and the first 300 amino acid sites of reverse transcriptase, corresponding to positions 2253–3500 of the international reference strain HXB2) was amplified by reverse transcription PCR and nested PCR, and the primer design was based on reference [20]. The PCR products were sent to Shanghai Sangon Biological Engineering Co., Ltd. (Shanghai, China) for Sanger sequencing.

### 2.3. Sequence Processing

Sequencher 5.4.6 was used to splice the sequences; MEGA X was used to correct the spliced sequences; COMET [21], HIV Blast [22] and phylogenetic tree were used for subtype analysis of the sequences. Surveillance drug resistance mutations (SDRMs) and drug resistance were analyzed using HIV Drug Resistance Database (hivdb.stanford.edu). SDRMs were designed to be sensitive and the specific indicators of antiretroviral drug selection pressure, compared with drug resistance mutations: (a) they are commonly recognized as causing or contributing to resistance; (b) they are nonpolymorphic in untreated persons; and (c) they are applicable to all HIV-1 subtypes [23]. The R package ape was used to calculate the pairwise gene distance, and the calculation model was TN93 [24].

The molecular transmission network was constructed by screening molecular pairs with gene distance ≤0.005, and the molecular transmission was observed. Samples associated within the molecular propagation network are connected by line segments, independent graphical units are molecular clusters, and the number of samples connected by a single sample is the degree value. The molecular cluster size of ≥5 samples was defined as a large molecular cluster, and the samples with degree rank in the top 25% (degree ≥4 in this study) were defined as active samples. Sample inclusion rate was calculated by samples in network/all samples. If a certain group has a high inclusion rate, it means that this group is highly likely to enter the molecular network, and the virus transmission within this group is also intensive.

### 2.4. Statistical Analysis

R 4.4.2 was used for statistical analysis. The count data were expressed as the number of cases (constituent ratio), and the comparison between groups was analyzed by χ^2^ test. *p* < 0.05 was considered statistically significant. Multiple logistic regression was used to calculate odds ratios (ORs) and 95% confidence intervals.

## 3. Results

### 3.1. Demographic Information on Populations

From 2020 to 2023, a total of 4235 people were newly diagnosed with HIV in Hangzhou. Among them, 3615 people with successfully obtained sequences were included in this study, including 1583 PMP cases (43.79%), 1172 TMP cases (32.42%) and 860 NMP cases (23.79%), achieving a sample coverage of 85.36%. The household registrations of migrant populations, in the descending order of frequency, were Zhejiang Province, Anhui Province, Henan Province, Jiangxi Province, Guizhou Province, and other provinces with fewer cases (Table 1).

As shown in Table 1, all three groups were predominantly male, and TMP had the lowest proportion of male (86.35%), while PMP had the highest (94.06%). Most people were 16–40 years old, with PMP having the highest proportion in this range (77.57%). Regarding occupation, PMP had the highest proportion of business service staff (47.06%) and students (5.69%); TMP had the highest proportion of workers (17.24%), farmers (21.42%), and domestic service staff (15.61%); NMP had the highest proportion of employees (10.70%). Regarding marital status, PMP had the highest proportion of unmarried people (71.51%), while over 30% of both TMP and NMP were married. Regarding education, PMP had the highest proportion of people with college and above degrees (47.19%); TMP accounted for the highest proportion with junior high school education or below (54.27%). Regarding sample sources, PMP accounted for the highest proportion of voluntary counseling and testing (VCT, 29.37%) and sexually transmitted disease (STD) clinic (20.34%) sources. Regarding sexual experiences, PMP accounted for the highest proportion reporting only homosexual experiences (73.66%), TMP accounted for the highest proportion reporting only heterosexual experiences (43.17%), and NMP accounted for the highest proportion reporting both homosexual and heterosexual experiences (7.21%). PMP had the highest proportion with an STD history (20.09%). Regarding the infectious route, PMP accounted for the highest proportion of people transmitted through homosexual contact (78.08%), while TMP and NMP had higher proportions of people transmitted through heterosexual contact than PMP. NMP accounted for the highest proportion of people diagnosed at the early (5.81%) and late (37.79%) stages. All reported results were statistically significant (*p* < 0.05).

### 3.2. Drug Resistance and Subtype

The surveillance drug resistance mutation rate among confirmed cases from 2020 to 2023 was 3.87% (140/3615), and the pretreatment drug resistance rate was 8.63% (312/3615). The major drug resistance mutations in drug resistance viruses were Q58E in the protease region and S68G/N, M14V/I, K103E/N/S, E138A/G/K/Q, and V179D/E/L/T in the revertase region. No significant difference in drug resistance was observed among the three populations (Table S1). A total of 23 circulating recombinant forms (CRFs) and multiple unique recombinant forms (URFs) were identified. The main subtypes for PMP were CRF07_BC (48.07%), CRF01_AE (35.69%), and CRF55_01B (4.80%); for TMP, they were CRF07_BC (44.37%), CRF01_AE (31.06%), CRF08_BC (7.85%), and CRF55_01B (4.27%); and for NMP, they were CRF07_BC (44.30%), CRF01_AE (34.19%), and CRF08_BC (5.58%). The differences in viral subtypes were statistically significant (*p* < 0.001, Table 1).

### 3.3. Molecular Transmission

**Figure 1:**
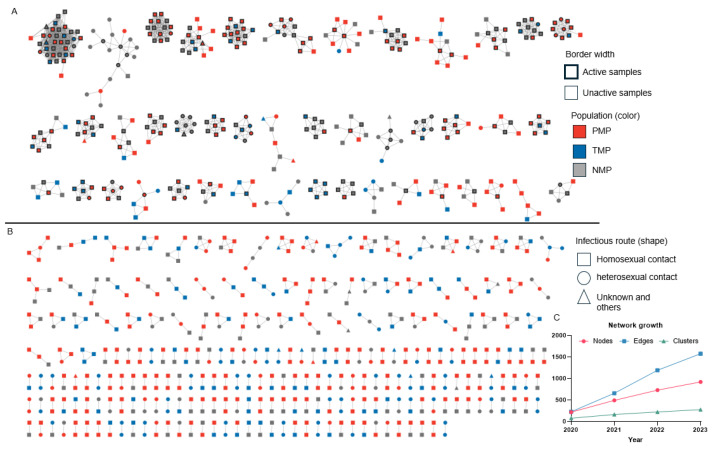
Molecular transmission network among migrant populations in Hangzhou. Sample pairs with gene distances less than 0.005 were included. A total of 833 samples formed 275 molecular clusters. Active samples (degree ≥4) are indicated with bold borders. (**A**) Molecular clusters with size ≥5, (**B**) molecular clusters with size <5, and (**C**) annual growth of the network from 2020 to 2023.

The molecular transmission network was constructed using a genetic distance threshold of <0.005 (Figure 1), and it included 833 people forming 275 molecular clusters, with a sample inclusion rate of 23.04%. The network comprised 767 males and 66 females, with 610 cases infected through homosexual contact and 201 through heterosexual contact. Inclusion rates for PMP, TMP and NMP were 27.04%, 18.94% and 21.28%, respectively. All molecular clusters involved migrant populations, PFP were present in 78.5% of molecular clusters (216/275), and TMP in 54.5% of molecular clusters (150/275). Network growth rate decreased annually, with node increase rates of 126%, 48% and 26% in 2021, 2022 and 2023, respectively (Figure 1C).

The molecular network obtained 44 large molecular clusters, with the largest molecular cluster containing 35 cases. There were 219 active samples, and the highest degree value was 32. The proportion of active samples was 35.16% for NMP, 26.17% for PMP, and 19.28% for TMP. The proportion of samples located in large molecular clusters was 51.65% for NMP, 39.72% for PMP and 28.25% for TMP. These differences were significant (Table 1). The frequency of connections between the groups from the highest to the lowest, was PMP-NMP (449), PMP-PMPs (355), PMP-TMP (260), NMP-NMPs (211), TMP-NMP (210), and TMP-TMP (91).

**Figure 2:**
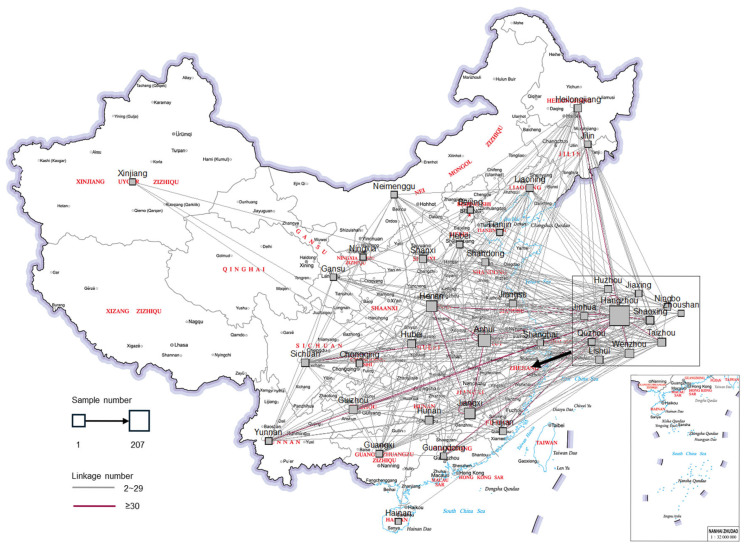
Geographical distribution of household registration for samples within the network (shown in Figure 1). The blocks indicate provinces (outside Zhejiang Province) or cities (inside Zhejiang Province). The lines indicate linkages between two locations. This map is based on a standard map (review number GS (2019) 1686) from the Standard Map Service Website of the Map Technical Review Center of the Ministry of Natural Resources. The base layer is not modified.

We analyzed the geographical distribution of household registration for samples within the molecular transmission network and present its map in Figure 2. Outside Zhejiang Province, many samples were from Anhui, Jiangxi, and Henan Provinces. Inside Zhejiang Province, most samples were from Hangzhou. Correspondingly, the most frequent inter-regional links were Hangzhou–Hangzhou, Anhui–Hangzhou, Jiangxi–Hangzhou, and Henan–Hangzhou.

To identify factors associated with network inclusion among migrants, multivariable logistic regression was performed with network entry as the dependent variable (Table 2). The cases that were easier to enter the network had higher inclusion rates. The results showed that cases with current residences in Hangzhou had higher inclusion rates than in others. Cases with household registration in Zhejiang Province had a higher inclusion rate than outside Zhejiang Province. Population with unknown STD history had a lower inclusion rate than population with STD history. Cases from VCT had higher inclusion rates than cases from other clinical sources and other sources. Cases at the late stage of diseases had a lower inclusion rate than the early stage.

## 4. Discussion

Hangzhou, as a central city in the Yangtze River Delta, experiences substantial population mobility, and migrant populations constitute 76.19% of people living with HIV, with PMP and TMP outnumbering NMP. The three populations showed significant differences in both demographic and molecular epidemiological characteristics. In this study, all the samples successfully obtained viral sequences, indicating high viral load infectiousness. This study reflects the ongoing HIV epidemic in Hangzhou and analyzes prevalent characteristics across populations, holding implications for targeted prevention. However, we have limitations in the method to detect accurate viral subtype, which only detect the pol gene. Moreover, phylogenetic analysis would somewhat be different from the real world and cannot reflect the direction of transmission. All participants of this study remain anonymous, and refusal by participants would not result in any consequences. Newly infected people received antiviral therapy upon their own request.

PMP constitutes nearly 40% of permanent residents in Zhejiang Province [25]. This population typically consists of formally employed individuals with stable work and residence, mostly of working age and highly educated, contributing significantly to regional development. These characteristics suggest PMP is more open-minded and adept at utilizing online platforms to seek same-sex sexual partners. The highest proportion of the CRF55_01B subtype in PMP may be related to this group’s higher rate of homosexual behavior, consistent with previous reports that this subtype primarily circulated among MSM populations [26].

We found PMP had relatively high self-testing awareness, as indicated by their highest proportions from VCT and STD. This likely contributed to their lower proportion of late-stage diagnoses. Motivation for self-testing among PMP may stem from partner notifications or personal hindsight. However, their higher education level is associated with a greater use of anonymous online platforms for partner-seeking, which can complicate contact tracing and delay testing, resulting in a lower early-diagnosis proportion compared to NMP. Further health promotion via new media targeting this unmarried, highly educated group is needed to enhance their protective behaviors and self-testing awareness.

TMP exhibits higher mobility than PMP, and they mainly comprise temporary visitors to Hangzhou and informal workers without local social insurance. Hangzhou’s robust healthcare system and cross-regional medical insurance policies attract patients from other regions [27]. The willingness of temporary visitors to seek medical care in Hangzhou is also strong; therefore, people living elsewhere had tested positive for HIV in Hangzhou. Informal workers are characterized by irregular hours, diverse workplaces, variable pay, informal labor relations, and weak social security, resulting in lower social integration than formal workers [28,29]. Although they are persistently living in Hangzhou like PMP, it is more difficult to track sexual partners and conduct testing for informal workers.

Homosexual contact remained the primary transmission route for TMP. This group of men who have sex with men (MSM) was difficult to track due to the prevalence of anonymous online platforms and high mobility. The molecular transmission network in Hangzhou was mainly composed of the MSM population, and utilizing this network can help identify sexual partners within the MSM population, reducing the difficulty of sexual partner tracking. To lower the incidence of TMP, efforts can focus on informal workers, providing humanitarian care tailored to their high working hours and low social security, alleviating psychological pressure, and effectively promoting awareness.

Following infection, HIV continuously mutates, increasing genetic distance between donor and recipient strains. HIV pro/rt diverges from the founder strain at a rate of about 0.1~0.2% per site per year. A threshold of 0.005 was recommended to identify molecular transmission relationships within about 3 years. This threshold can sensitively identify recent links and avoid false links [30,31]. The primary inter-regional links observed were from Jiangxi, Anhui, and Henan Provinces to Hangzhou, suggesting that migrants from these regions are deeply involved in local transmission. This pattern extends beyond Hangzhou; our previous study found genetic similarities between sequences from Hangzhou and those from other major economic hubs such as Shenzhen, Beijing, and Guangzhou [32]. Collectively, these cities are key migration destinations due to their economic development. The data presented in Figure 2, combined with these genetic links, support the hypothesis that migrants from developing provinces such as Jiangxi, Anhui and Henan contribute significantly to HIV transmission across multiple economically developed cities. Several larger molecular clusters included cases infected via both homosexual and heterosexual contacts, the latter primarily being men. This suggesting that some individuals may not disclose heterosexual behavior during investigations or may have undisclosed female partners.

The deep participation of PMP in social and economic activities was accompanied by the local transmission of HIV. Over half of samples in the molecular transmission network were of PMP, and most molecular associations also involved PMP. TMP is present in over half of molecular clusters, and primarily linked to PMP, suggesting they bridge multiple intercity and inter-provincial transmission chains. NMP had the highest proportion of active samples, and the highest presence in large molecular clusters, suggesting that it could be serve as starting points for contact tracing, potentially extending to migrant contacts.

Logistic regression analysis indicated that TMP residing in other cities within Zhejiang Province had the lowest network inclusion rate, possibly because their social and sexual networks remain centered outside Hangzhou. Cases with STD testing histories had stronger self-testing awareness, making them more likely to be found in the networks. Due to the long infection period, it was difficult to find people that were associated with late-stage-diagnosed people. Late-stage-diagnosed people may acquire HIV outside Hangzhou. To trace their sexual partners outside Hangzhou, we recommend improving intercity cooperation and information sharing.

In summary, most people living with HIV in Hangzhou are migrant populations, who played a major role in ongoing HIV transmission. PMP entails young and highly educated people, primarily comprising the MSM population, and serves as the main driver of local transmission, forming large molecular clusters with NMP. TMP has complex origins, with a relatively high proportion infected through heterosexual contact, playing an important role in viral flow. Informal workers in TMP are a key target for intervention. To reduce undiagnosed infection and transmission, targeted health promotion for unmarried, highly educated individuals and informal workers should emphasize condom use, correct post-exposure prophylaxis, and timely self-testing. The use of molecular transmission networks can help uncover transmission chains, optimize resource allocation, and address the challenges of rapid intercity and inter-provincial viral mobility.

## Figures and Tables

**Table 1:** Demographic and molecular epidemiological information of migrant populations.

Variables	Permanent Migrant Population (Case/%)	Temporary Migrant Population (Case/%)	Non-Migrant Population (Case/%)	Chi-Square Value	*p* Value
Number of cases	1583	1172	860		
Household registration (province)				29.9	<0.001
Zhejiang	339 (21.42)	267 (22.78)	860 (100.00)		
Anhui	204 (12.89)	146 (12.46)			
Henan	158 (9.98)	106 (9.04)			
Jiangxi	124 (7.83)	116 (9.90)			
Guizhou	51 (3.22)	82 (7.00)			
Others	707 (44.66)	455 (38.82)			
Sex				47.63	<0.001
Male	1489 (94.06)	1012 (86.35)	771 (89.65)		
Female	94 (5.94)	160 (13.65)	89 (10.35)		
Age groups				269.2	<0.001
16–40	1228 (77.57)	643 (54.86)	453 (52.67)		
41–59	309 (19.52)	421 (35.92)	270 (31.4)		
>60	46 (2.91)	108 (9.22)	137 (15.93)		
Occupations				319.3	<0.001
Business service staff	745 (47.06)	373 (31.83)	250 (29.07)		
Worker	249 (15.73)	202 (17.24)	124 (14.42)		
Farmer	94 (5.94)	251 (21.42)	150 (17.44)		
Domestic service staff	125 (7.9)	183 (15.61)	83 (9.65)		
Employee	132 (8.34)	30 (2.56)	92 (10.70)		
Student	90 (5.69)	35 (2.99)	37 (4.30)		
Others	148 (9.35)	98 (8.36)	124 (14.42)		
Marital status				249.6	<0.001
Unmarried	1132 (71.51)	548 (46.76)	391 (45.47)		
Married	235 (14.85)	386 (32.94)	302 (35.12)		
Divorced	195 (12.32)	223 (19.03)	157 (18.26)		
Unknown	21 (1.33)	15 (1.28)	10 (1.16)		
Education				320.2	<0.001
Illiterate	16 (1.01)	31 (2.65)	23 (2.67)		
Primary school	98 (6.19)	222 (18.94)	131 (15.23)		
Junior high school	308 (19.46)	383 (32.68)	174 (20.23)		
Senior high school	414 (26.15)	315 (26.88)	192 (22.33)		
College and above	747 (47.19)	221 (18.86)	340 (39.53)		
Source of sample				141.3	<0.001
VCT	465 (29.37)	202 (17.24)	148 (17.21)		
STD clinic	322 (20.34)	177 (15.1)	102 (11.86)		
Other clinical samples	742 (46.87)	739 (63.05)	582 (67.67)		
Others	54 (3.41)	54 (4.61)	28 (3.26)		
Sexual experiences				244.4	<0.001
Only homosexual	1166 (73.66)	582 (49.66)	439 (51.05)		
Only heterosexual	300 (18.95)	506 (43.17)	331 (38.49)		
With both men and women	72 (4.55)	37 (3.16)	62 (7.21)		
Unknown	45 (2.84)	47 (4.01)	28 (3.26)		
Presence of STD history				17.19	0.002
Yes	318 (20.09)	182 (15.53)	150 (17.44)		
No	1174 (74.16)	887 (75.68)	644 (74.88)		
Unknown	91 (5.75)	103 (8.79)	66 (7.67)		
Infectious route				226.9	<0.001
Homosexual contact	1236 (78.08)	614 (52.39)	495 (57.56)		
Heterosexual contact	298 (18.83)	503 (42.92)	334 (38.84)		
Unknown	49 (3.10)	55 (4.69)	31 (3.60)		
Infectious stage at the time of diagnosis				23.5	<0.001
Early stage	82 (5.18)	52 (4.44)	50 (5.81)		
Medium stage	1042 (65.82)	729 (62.20)	485 (56.4)		
Late stage	459 (29.00)	391 (33.36)	325 (37.79)		
HIV subtype				50.99	<0.001
CRF07_BC	761 (48.07)	520 (44.37)	381 (44.3)		
CRF01_AE	565 (35.69)	364 (31.06)	294 (34.19)		
CRF08_BC	48 (3.03)	92 (7.85)	48 (5.58)		
CRF55_01B	76 (4.80)	50 (4.27)	34 (3.95)		
Others	133 (8.4)	146 (12.46)	103 (11.98)		
Whether to enter the molecular transmission network				26.87	<0.001
Yes	428 (27.04)	222 (18.94)	183 (21.28)		
No	1155 (72.96)	950 (81.06)	677 (78.72)		
Whether was an active sample ^1^				13.05	0.001
Yes	112 (26.17)	43 (19.28)	64 (35.16)		
No	316 (73.83)	180 (80.72)	118 (64.84)		
Whether in large molecular cluster ^2^				23.09	<0.001
Yes	170 (39.72)	63 (28.25)	94 (51.65)		
No	258 (60.28)	160 (71.75)	88 (48.35)		

Notes: ^1^ This variable only counted samples which enter the molecular transmission network, and samples whose degree was ≥4 were active samples; ^2^ this variable only counted samples which enter the molecular transmission network, and molecular clusters with a size ≥5 were large molecular clusters. Abbreviations: VCT, voluntary counseling and testing; STD, sexually transmitted disease.

**Table 2:** Factors of migrant populations entering the molecular transmission network.

Variables	Cases	Cases Included in Network (Inclusion Rate, %)	Chi-Square Value	*p* Value	aOR Value (95%CI)	*p* Value
Current residence			24.98	<0.001		
Hangzhou	1583	428 (27.04)			1.000	
Zhejiang Province except Hangzhou	336	59 (17.56)			0.548 (0.39~0.769)	0.001
Outside Zhejiang Province	836	163 (19.50)			0.789 (0.636~0.979)	0.031
Household registration			5.692	0.017		
In Zhejiang Province	606	165 (27.23)			1.000	
Outside Zhejiang Province	2149	485 (22.57)			0.662 (0.519~0.845)	0.001
Presence of STD history			16.09	<0.001		
Yes	500	139 (27.80)			1.000	
No	2061	485 (23.53)			0.877 (0.699~1.101)	0.258
Unknown	194	26 (13.40)			0.443 (0.278~0.706)	0.001
Source of sample			43.83	<0.001		
VCT	667	208 (31.18)			1.000	
STD clinic	499	138 (27.66)			0.841 (0.647~1.093)	0.196
Other clinical samples	1481	286 (19.31)			0.6 (0.484~0.744)	<0.001
Others	108	18 (16.67)			0.441 (0.258~0.754)	0.003
Infectious stage at diagnosis			21.52	<0.001		
Early stage	134	37 (27.61)			1.000	
Medium stage	1771	460 (25.97)			0.823 (0.541~1.252)	0.363
Late stage	850	153 (18.00)			0.542 (0.349~0.843)	0.009

Notes: The dependent variable was entry into the molecular transmission network. Only independent variables significant in the multivariable model are shown. Independent variables included age, current residence, household registration, infectious route, sexual experiences, presence of STD history, source of sample, marital status, occupations, education and infectious stage at diagnosis. All were significant in univariate Chi-square test. No multicollinearity was detected (tolerance: 0.312~0.980; variance inflation factor values: 1.039~3.208).

## Data Availability

The datasets used and analyzed during the current study are available from the corresponding author on reasonable request.

## Supplementary Materials

The following supporting information can be downloaded at: https://www.mdpi.com/article/10.3390/v18030365/s1. Table S1: Major drug resistance mutations in drug-resistant people.

## Abbreviations

PMP	Permanent migrant population
TMP	Temporary migrant population
NMP	Non-migrant population
SDRMs	Surveillance drug resistance mutations
OR	Odds ratio
STD	Sexually transmitted disease
VCT	Voluntary counseling and testing
MSM	Men who have sex with men
