# Supplementary material for: Molecular Transmission Dynamics of HIV-1 in Migrant Populations: Transmission Clusters and Demographic Diversity in Hangzhou, a Key Migration Hub in Eastern China
Source: Viruses. 2026 Mar 16;18(3):365. doi: 10.3390/v18030365 (PMC13030782; doi:10.3390/v18030365)
Supplement: Supplementary file 1 [file viruses-18-00365-s001.zip › viruses-4178367-supplementary.pdf]

Table S1 Drug resistance mutations in drug resistant viruses

	PMP	TMP	NMP	Chi-square value	*p* value
Drug resistant viruses	124	122	66		
Viruses resistant to PI	39	44	18	1.592	0.451
Q58E	28	30	10	1.502	0.472
Viruses resistant to NRTI	20	17	14	1.666	0.435
S68G/N	8	7	7	0.376	0.829
M184V/I	6	7	5	0.504	0.777
Viruses resistant to NNRTI	71	67	36	0.187	0.911
K103E/N/S	31	23	12	1.683	0.431
E138A/G/K/Q	23	22	14	0.506	0.777
V179D/E/L/T	20	15	8	0.771	0.68

Note: Chi-square test was used for comparison.
